# Supplementary material for: Activation of AMPD2 drives metabolic dysregulation and liver disease in mice with hereditary fructose intolerance
Source: Commun Biol. 2024 Jul 11;7:849. doi: 10.1038/s42003-024-06539-1 (PMC11239681; doi:10.1038/s42003-024-06539-1)
Supplement: Supplementary file 2 — Supplemental Figure 1 [file 42003_2024_6539_MOESM2_ESM.docx]

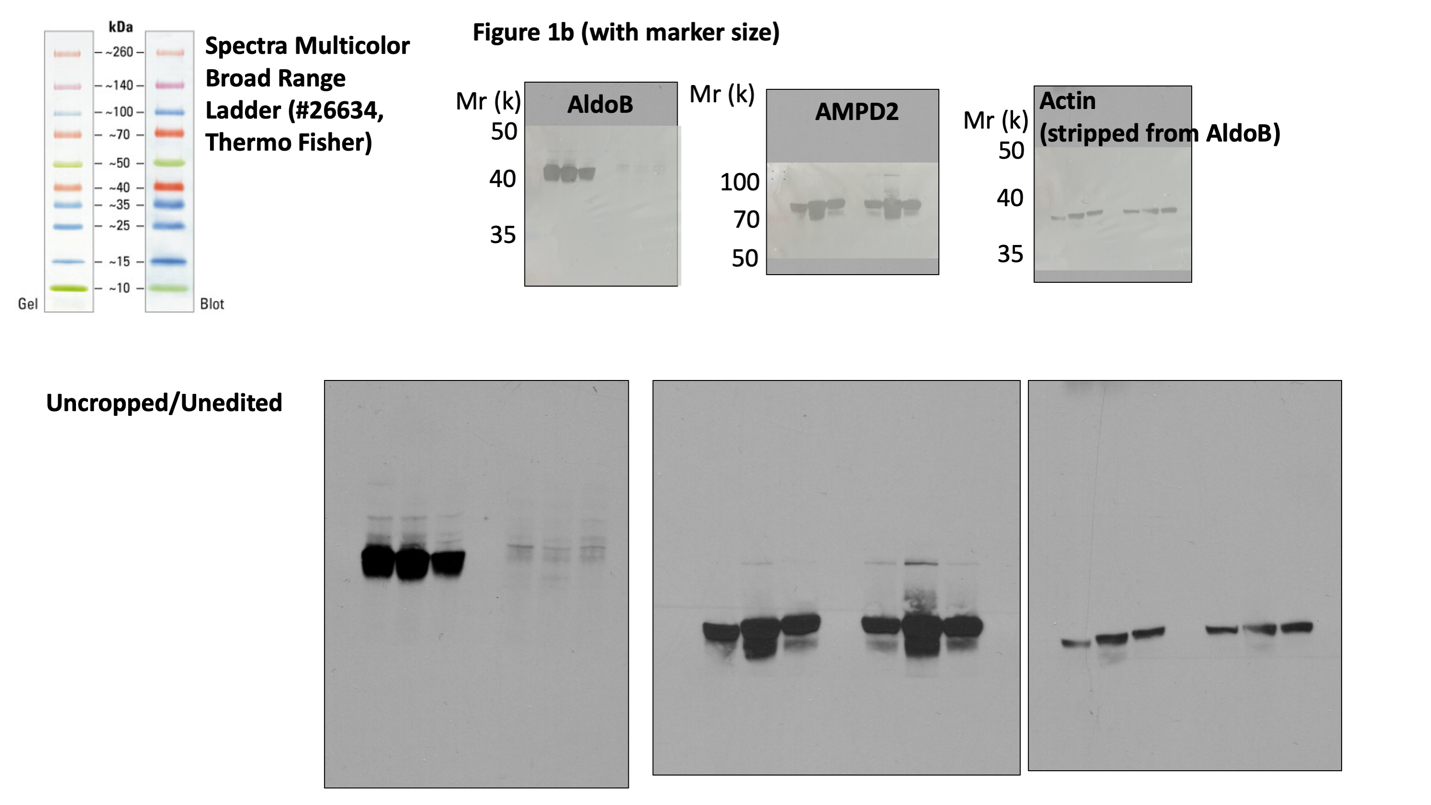


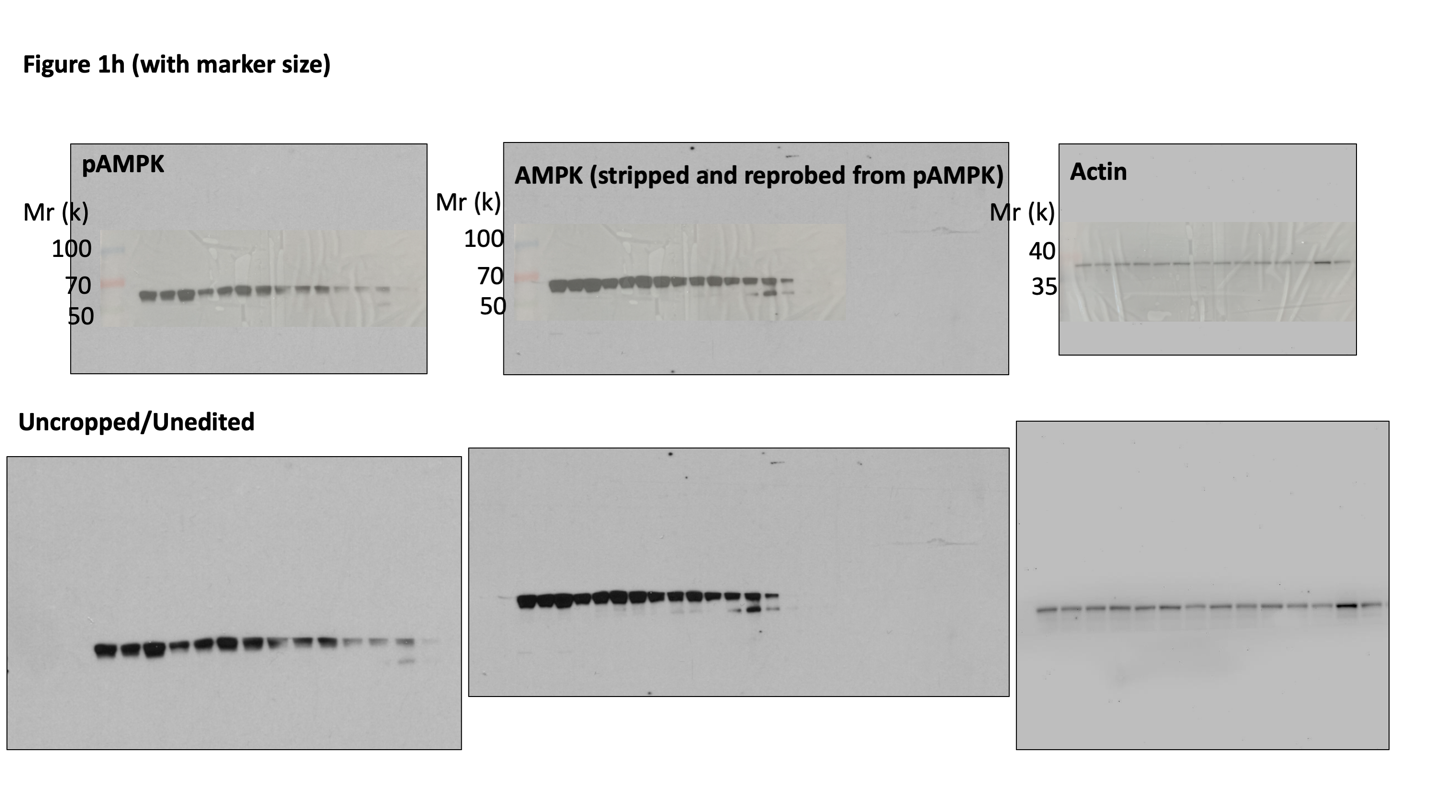


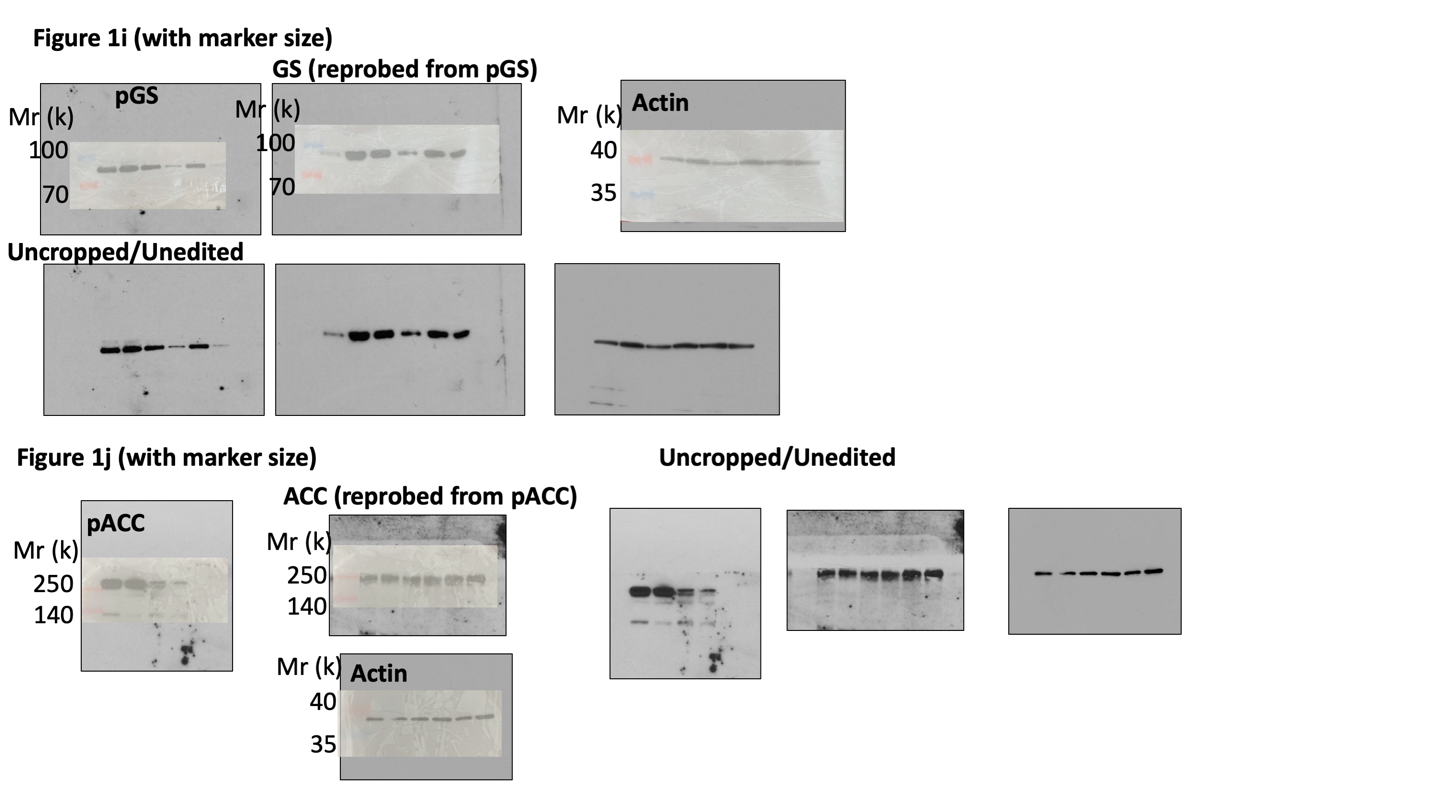


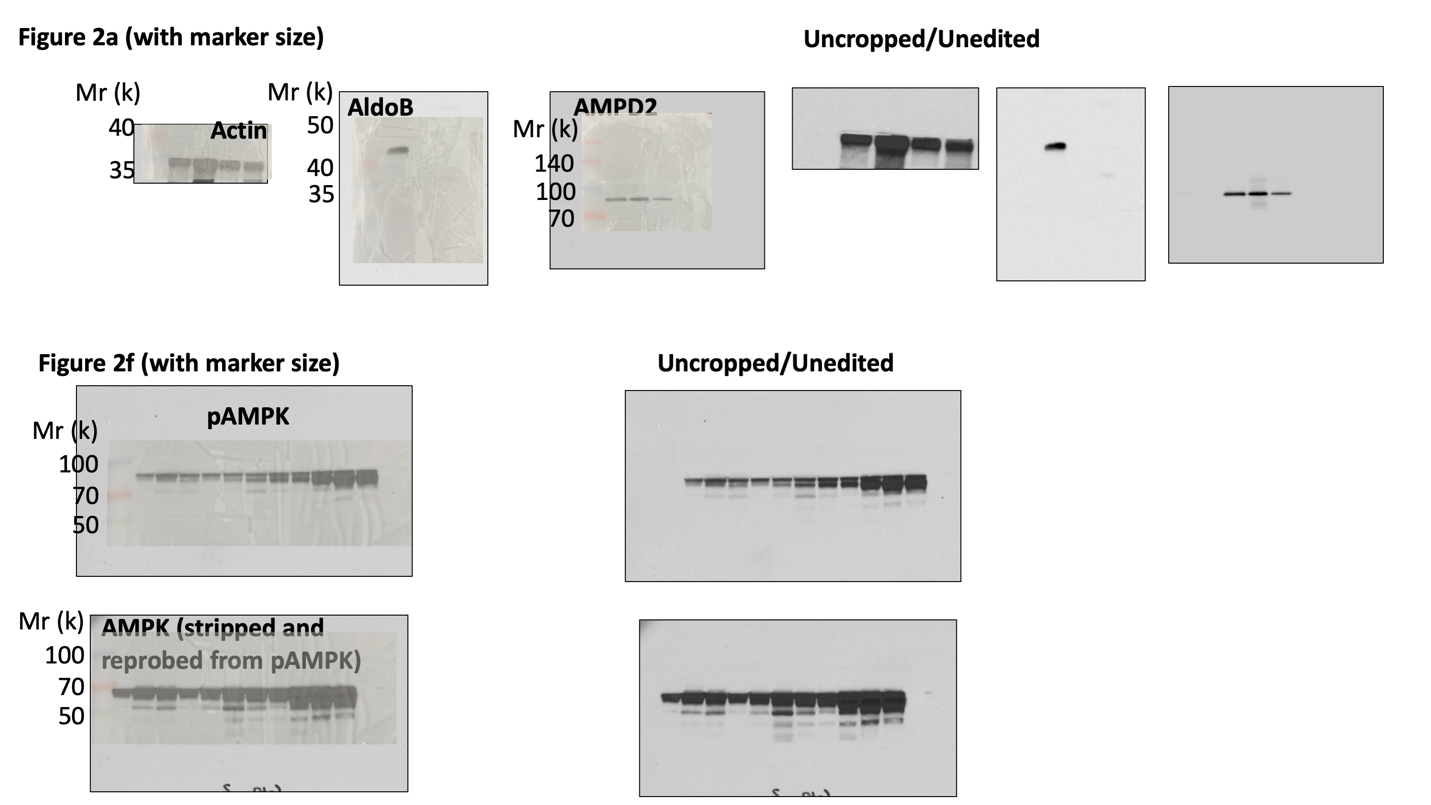


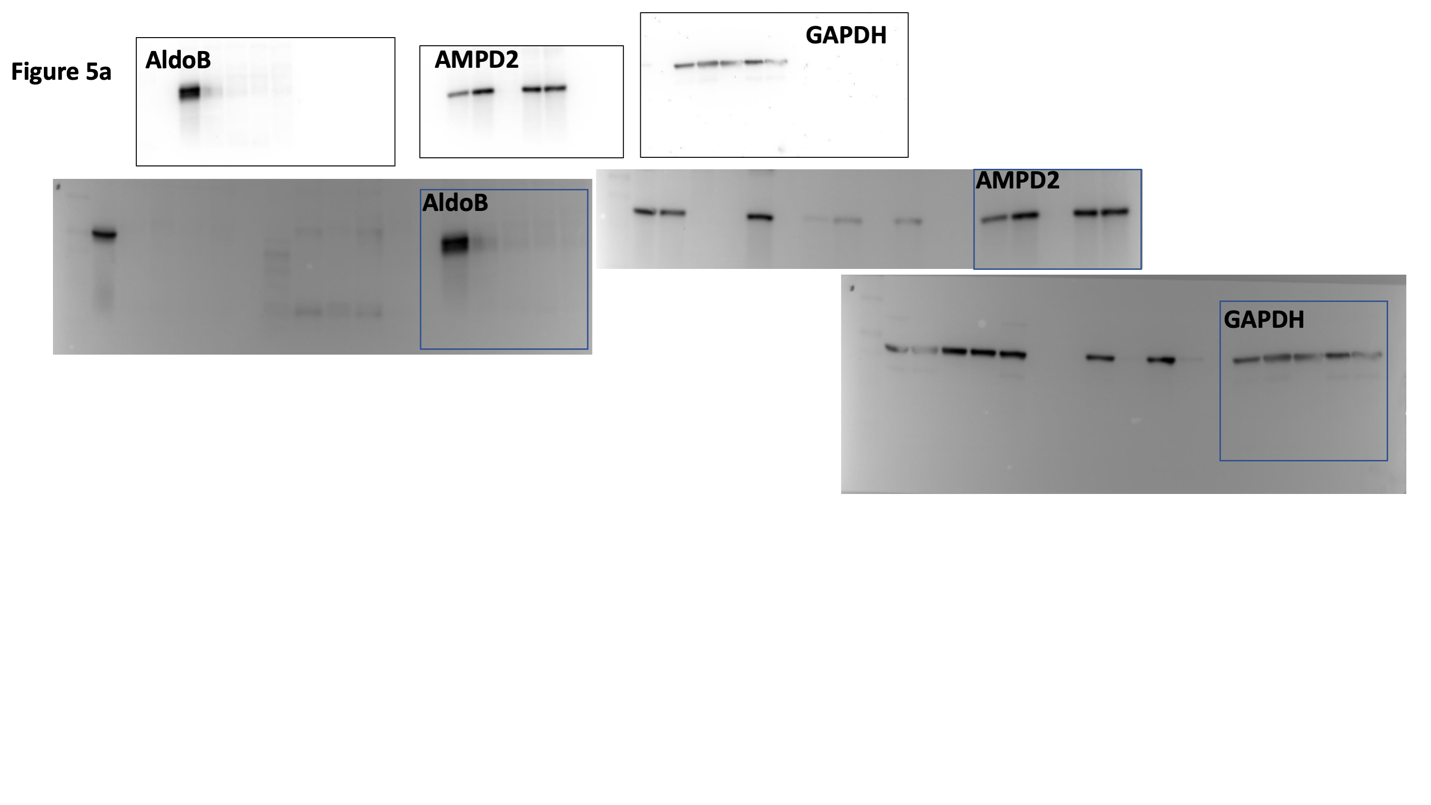


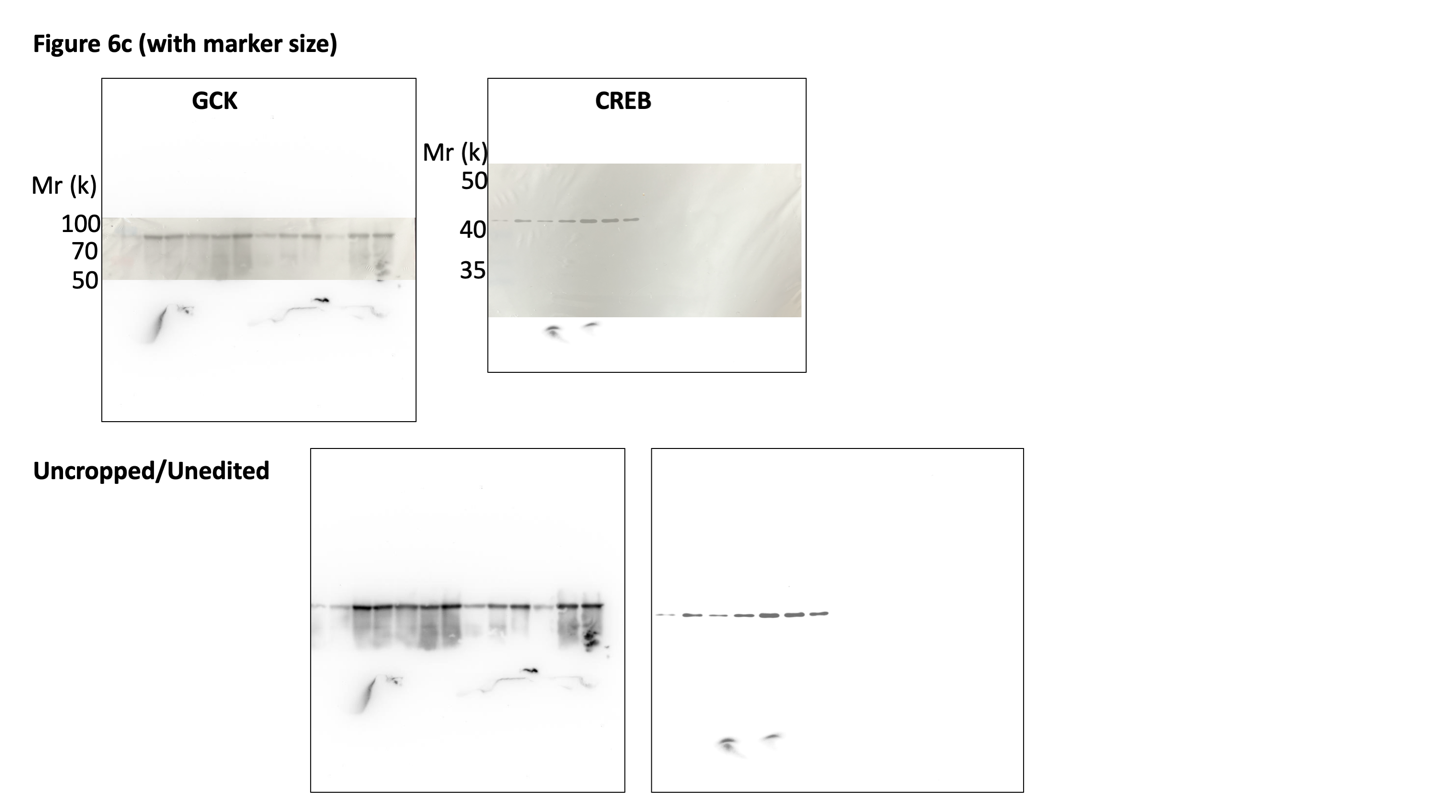


Supplementary Figure 1. Uncropped blots and reference markers identified by co-exposure of film and membrane (Figs. 1,2 and 6) or by chemical analysis with a Jena autoanalyzer (Fig. 5)
