# Supplementary material for: Activation of AMPD2 drives metabolic dysregulation and liver disease in mice with hereditary fructose intolerance
Source: Commun Biol. 2024 Jul 11;7:849. doi: 10.1038/s42003-024-06539-1 (PMC11239681; doi:10.1038/s42003-024-06539-1)
Supplement: Supplementary file 5 — Reporting Summary [file 42003_2024_6539_MOESM5_ESM.pdf]

nature portfolio

Corresponding author(s): Miguel A. Lanaspa

Last updated by author(s): Dec 19, 2023

Reporting Summary

Nature Portfolio wishes to improve the reproducibility of the work that we publish. This form provides structure for consistency and transparency in reporting. For further information on Nature Portfolio policies, see our Editorial Policies and the Editorial Policy Checklist.

Statistics

For all statistical analyses, confirm that the following items are present in the figure legend, table legend, main text, or Methods section.

n/a	Confirmed	
☐	☒	The exact sample size (*n*) for each experimental group/condition, given as a discrete number and unit of measurement
☐	☒	A statement on whether measurements were taken from distinct samples or whether the same sample was measured repeatedly
☐	☒	The statistical test(s) used AND whether they are one- or two-sided<br>*Only common tests should be described solely by name; describe more complex techniques in the Methods section.*
☐	☒	A description of all covariates tested
☒	☐	A description of any assumptions or corrections, such as tests of normality and adjustment for multiple comparisons
☐	☒	A full description of the statistical parameters including central tendency (e.g. means) or other basic estimates (e.g. regression coefficient) AND variation (e.g. standard deviation) or associated estimates of uncertainty (e.g. confidence intervals)
☒	☐	For null hypothesis testing, the test statistic (e.g. *F*, *t*, *r*) with confidence intervals, effect sizes, degrees of freedom and *P* value noted<br>*Give P values as exact values whenever suitable.*
☒	☐	For Bayesian analysis, information on the choice of priors and Markov chain Monte Carlo settings
☒	☐	For hierarchical and complex designs, identification of the appropriate level for tests and full reporting of outcomes
☒	☐	Estimates of effect sizes (e.g. Cohen's *d*, Pearson's *r*), indicating how they were calculated

Our web collection on statistics for biologists contains articles on many of the points above.

Software and code

Policy information about availability of computer code

Data collection	No software was used
Data analysis	Data graphics and statistical analysis were performed using Prism 7 (GraphPad).

For manuscripts utilizing custom algorithms or software that are central to the research but not yet described in published literature, software must be made available to editors and reviewers. We strongly encourage code deposition in a community repository (e.g. GitHub). See the Nature Portfolio guidelines for submitting code & software for further information.

Data

Policy information about availability of data

All manuscripts must include a data availability statement. This statement should provide the following information, where applicable:

- Accession codes, unique identifiers, or web links for publicly available datasets
- A description of any restrictions on data availability
- For clinical datasets or third party data, please ensure that the statement adheres to our policy

Data and Materials availability: Further information and requests for resources and reagents should be directed and will be fulfilled by the Lead Contact, Miguel A. Lanaspa (Miguel.lanaspagarcia@cuanschutz.edu). Mouse lines generated in this study are available for any researcher upon reasonable request. Similarly, all data reported in this paper will be shared by the lead contact upon request.This paper does not report original code. Any additional information required to reanalyze the data reported in this paper is available from the lead contact upon request

Research involving human participants, their data, or biological material

Policy information about studies with human participants or human data. See also policy information about sex, gender (identity/presentation), and sexual orientation and race, ethnicity and racism.

Reporting on sex and gender	N/A
Reporting on race, ethnicity, or other socially relevant groupings	N/A
Population characteristics	N/A
Recruitment	N/A
Ethics oversight	N/A

Note that full information on the approval of the study protocol must also be provided in the manuscript.

Field-specific reporting

Please select the one below that is the best fit for your research. If you are not sure, read the appropriate sections before making your selection.

☒ Life sciences ☐ Behavioural & social sciences ☐ Ecological, evolutionary & environmental sciences

For a reference copy of the document with all sections, see nature.com/documents/nr-reporting-summary-flat.pdf

Life sciences study design

All studies must disclose on these points even when the disclosure is negative.

Sample size	An a priori power analysis was not performed for determining sample size, but instead prior experience with the acute and chronic fructose-exposure of aldob(-/-) mouse model was used to establish a sample size sufficient to delineate therapeutic efficacy.
Data exclusions	no mice were excluded from the study due to health concerns or any other criterion.
Replication	When indicated, number of replicates were employe to ensure reproducibility of our findings.
Randomization	Mice were randomly assigned to the dietary regimens using the Random function in Excel and all samples/slides were analyzed in a blinded fashion and then decoded, grouped and the results analyzed.
Blinding	Mice were randomly assigned to the dietary regimens using the Random function in Excel and all samples/slides were analyzed in a blinded fashion and then decoded, grouped and the results analyzed.

Reporting for specific materials, systems and methods

We require information from authors about some types of materials, experimental systems and methods used in many studies. Here, indicate whether each material, system or method listed is relevant to your study. If you are not sure if a list item applies to your research, read the appropriate section before selecting a response.

Materials & experimental systems

n/a	Involved in the study	
☐	☒	Antibodies
☒	☐	Eukaryotic cell lines
☒	☐	Palaeontology and archaeology
☐	☒	Animals and other organisms
☒	☐	Clinical data
☒	☐	Dual use research of concern
☒	☐	Plants

Methods

n/a	Involved in the study	
☒	☐	ChIP-seq
☒	☐	Flow cytometry
☒	☐	MRI-based neuroimaging

Antibodies

Antibodies used	aldolase B (H00000229-A01), GCK (H000026465-B02P), ACC (3676), p-ACC (Ser79, 11818), AMPK (2603), pAMPK (Thr172, 2535), GS

Antibodies used	(3886), and pGS (Ser641 3981) (all from Cell Signaling); and AMPD2 (Abnova 271).
Validation	The majority of the antibodies employed were previously validated in our lab, some of them using samples from specific knockout mice for those antibodies as negative controls and published by our group before. Further, proof of specificity is provided by the company were the antibodies were purchased from which often were accompanied with references from peer-reviewed publications

Animals and other research organisms

Policy information about studies involving animals; ARRIVE guidelines recommended for reporting animal research, and Sex and Gender in Research

Laboratory animals	Mice, 8-10 weeks of age both male and female.
Wild animals	N/A
Reporting on sex	Animals from both sexes were included in the study and data pooled together for analysis.
Field-collected samples	N/A
Ethics oversight	All animal experiments were conducted with adherence to the National Institutes of Health Guide for the Care and Use of Laboratory Animals. The animal protocol (#1253, PI. Lanaspa) was approved by the Institutional Animal Care and Use Committee of the University of Colorado (Aurora, CO, USA).

Note that full information on the approval of the study protocol must also be provided in the manuscript.

Plants

Seed stocks	N/A
Novel plant genotypes	N/A
Authentication	N/A
